# En route to flourishing - a longitudinal mixed methods study of long-term unemployed citizens in an interdisciplinary rehabilitation program

**DOI:** 10.1186/s12889-022-13060-9

**Published:** 2022-04-07

**Authors:** Lotte Nygaard Andersen, Mette Jensen Stochkendahl, Kirsten K. Roessler

**Affiliations:** 1 Department of Sports Science and Clinical Biomechanics, University of Southern Denmark, Odense, Denmark; 2 Chiropractic Knowledge Hub, Odense, Denmark; 3 Department of Psychology, University of Southern Denmark, Odense, Denmark

**Keywords:** Work ability, Work skills, Survey, Qualitative interviews, Vocational rehabilitation, Social service interventions, PERMA model, Municipality

## Abstract

**Background:**

Interdisciplinary rehabilitation programmes (IRP) are used in municipality settings to assist unemployed citizens with complex health and/or life issues. Individually tailored IRP activities help people develop their personal working life skills and increase their chances of re-entering the work force. The aims of this paper were to describe citizens’ wellbeing in terms of health aspects, explore the impact of stressful life events on wellbeing and obtain understanding of how IRP activities affect the participants’ development towards future employment.

**Methods:**

A mixed methods exploratory approach has been used. For data collection a quantitative longitudinal survey (baseline and 1-year follow-up) and qualitative interviews were conducted. Descriptive statistics were used for the analysis of survey data, while the data material from interviews was analysed using directed content analysis. Results were discussed with the theory of flourishing as a framework to develop understanding.

**Results:**

At baseline, 146 respondents (71% females) filled in the survey and seven participants were interviewed. The analysis of survey data and interviews revealed five themes: (1) Stressful life events, (2) Positive emotions - how IRP-activities positively impacted wellbeing and physical capacity, (3) Appreciation of engagement, (4) Relationships, and (5) Meaning and optimal functioning. Results showed that IRP participants from the outset experienced high general pain intensity as well as distress, anxiety and depression. Life events relating both to physical health and work life were significant for their wellbeing. IRP activities supported participants’ positive development towards future employment in ways that were specific to each individual.

**Conclusions:**

From this study it can be derived that participants’ development took place around self-acceptance, acceptance by others, physical capacity, psychological resources and capacity to balance engagement to cultivate the best version of themselves. In future programmes, it may be emphasized that participants’ interest may be an important driver for wellbeing and future employment.

**Trial registration:**

ClinicalTrials.gov Identifier: NCT02641704, date of registration December 29, 2015.

## Background

In 2013, the Danish government launched a series of labour market policy reforms. Among these were a reform of disability pension and flexi-job schemes (light jobs supported by subsidies). The overall goals were to get as many citizens as possible into work and so enable them to provide for themselves and to ensure that as few citizens as possible end up on permanent disability pension [1]. Occupational rehabilitation teams in each of the Danish municipalities were established with the mandate to assign citizens to interdisciplinary rehabilitation programmes (IRP) consisting of activities based on the recommendation from the rehabilitation team and with the aim of assisting citizens in development towards employment [1].

IRP’s are typically designed with a focus on employment in the competitive labour market to help people develop their chances of re-entering the workforce [2, 3]. The intervention components are a combination of health-improving activities and job-related training. While they have been shown to have the potential to assist the unemployed and increase the number of employed people, the evidence is limited [4] and what there is suggests its effects are small [5, 6].

From 2014 to 2019, approximately 25–30% of all long-term unemployed persons embarked on IRP in Denmark. The target demographic for the programme were those with a complex set of problems for whom social-, employment- and health-related challenges created barriers to entering the labour market, putting them at risk of ending up on a permanent disability pension. In 2019, 5–7% of participants under 40 years of age and 1–3% of the + 40-year-old participants had gained regular, unsubsidized employment [7].

The rehabilitation teams assigning individuals to IRP are made up of representatives from employment, health care, social affairs, and educational sectors in the municipalities. On the basis of their combined expertise, they recommended IRP components individually tailored to each participant on the programme. This approach caters for participants with a complex set of problems [8] and ensures a holistic approach consisting of relevant activities across the various sectors represented in the team. The IRP was designed to last from one to five years and to help participants develop the individual skills needed to increase their chances of re-entering employment. Its aims are similar to other rehabilitation programmes such as Individual Placement and Support (IPS) [3, 9]. IPS involves individualized job support, a focus on competitive employment (e.g. internship programmes) and has previously been successful with vulnerable groups such as those suffering from severe mental disorders [3, 9]. The Danish IRP contain many of the same activities as IPS, but knowledge is scarce about how these types of programmes and the underlying processes involved can lead to employment [10]. Specifically, it is unclear how the simultaneous presence of physical and mental health problems set alongside social issues and employment challenges affect the outcome of IRP [10–12].

In 2017, Scott et al. [13] described the complex journey undertaken by those challenged by life events or serious illness towards healing and making a life that was worth living. They found that the routes taken depend on individual characteristics and on the nature and timing of the initial mental and/or physical damage [13]. The life events studied by Scott et al. [13] include those that induce stress such as divorce, the death of a spouse, loss of a job, illness etc. Healing includes individual flourishing, and having a job matters for healing and flourishing if it engages the individual and provides meaning [14, 15].

In this project, scrutinizing citizens’ participation in IRP in Sonderborg Municipality, positive psychology will be used as a theoretical framework for analysis. Positive psychology may focus on those aspects that make life worth living and contribute most to the prospect of a positive future and a flourishing and fulfilling life [16–19], but it also deals with the impact of stressful life events on wellbeing [15, 20, 21]. Flourishing was used as a psychological construct to explore, how participants cultivated the best version of themselves within their particular limitations and how IRP contributed to promoting the participants’ capacity to work [22]. The focus in the flourishing approach is on how to increase flourishing and learning to live a good life. The focus is not on mental illness, painful conditions, or negative life circumstances. Though, descriptions of participants involve illness and pain (aim 1), while investigation of processes relating to personal development as a result of IRP (aim 2 and 3) use a flourishing approach. Flourishing is a multidimensional construct made up of several important psychological elements [22]. The psychologist Martin Seligman has explained flourishing as a construct in the PERMA model consisting of five elements: **P**ositive emotions, **E**ngagement, **R**elationships, **M**eaning and purpose, and **A**ccomplishments [19, 22]. The PERMA model can guide individuals to find paths to a flourishing life [23]. To our knowledge, this is one of the first research projects using a mixed methods approach to investigate citizens’ participation in IRP in Denmark [12]. Combining quantitative and qualitative data with a framework inspired from psychology enables detailed descriptions of citizens in an IRP and deepen our understanding of the citizens’ complex situations and development.

This project’s overall aims are to describe characteristics of citizens assigned to IRP in Sonderborg Municipality in Denmark and understand the personal development they undergo as a result of their rehabilitation activities [12]. In this paper, results were discussed with the theory of flourishing as a framework to develop understanding and we use the PERMA model as a framework for analysis to explore the citizens’ experiences of IRP activities and their acquisition of individual skills to enable flourishing and make life worth living [19, 22].

Specifically we aim to: 1) describe participants’ wellbeing in terms of physical and mental health aspects at baseline and one-year follow-up (pain, depression, anxiety and loneliness), 2) explore the impact of stressful life events on participants’ wellbeing using the psychological construct of flourishing as a criterion, and 3) obtain an understanding of how IRP activities affect the participants’ development towards future employment using the PERMA model as an analytical framework.

## Methods

### Study design

A mixed methods exploratory approach [24] was used to provide a better understanding of the complex nature of the study aims regarding participation in IRP. The use of a mixed methods research methodology in this paper involved qualitative interviews and a quantitative longitudinal survey for data collection. Both data types were collected concurrently and analysed separately during the initial phase of analysis. Subsequently in the results section, the results of the analysis were combined and presented in an integrated design following the themes (described in “Interview analysis”). In the discussion the combined results are discussed and interpreted to generate insight into the study population, gain in-depth knowledge of the participants’ experiences and about significant IRP mechanisms. This combined design provides a better understanding than either alone could provide.

This study is part of a larger project, and a complete description of methods used for the project has previously been described in Andersen et al. [12].

The study was conducted in accordance with the WMA Declaration of Helsinki ethical principles [25]. The protocol was considered exempt from ethical approval by the Regional Scientific Ethics Committee for Southern Denmark as the study does not fall within the scope of the Medical Research Involving Human Subject Act (§14). SDU Research & Innovation Organization has approved the data management in the project as part of the common agreement with the Danish Data Protection Agency, ref. no. 15/96413. The project is registered in the ClinicalTrials.gov, number NCT02641704.

### Participants in IRP

Participants were long-term unemployed citizens enrolled in an IRP in Sonderborg Municipality, Denmark. Criteria for enrollment into the IRP programmes were defined by the Danish Ministry of Labour in the following way [1, 26]:

- being unemployed and aged 18 to 65 years,
- being at high risk for permanent disability pension because of complex challenges involving social, employment and/or health problems,
- not being ready to enter the labour market and requesting continued education or retraining of skills due the complex nature of their problems,

### Interdisciplinary rehabilitation programme (IRP)

At a national level, the overall aims and content of IRP are uniform and aimed at enhancing the citizen’s work ability [1]. The municipal rehabilitation team made use of a holistic approach when they put together the programme. The programme included individually tailored activities either as single components or in combination. Examples of activities included training in social and/or work skills (e.g. internship), self-management courses (e.g. related to depression or overweight) and individual meetings with a coordinator. A coordinator was assigned to each citizen and supported the citizen in carrying out the programme.

### Qualitative interview procedures

Interview participants were selected with a purposeful strategy aiming at including interview participants providing in-depth information for answering our study aims [27, 28]. We aimed for in-depth information and not for generalised answers. That entailed that interview participants were recruited stepwise, they had varying characteristics in relation to sex, age, time into VRP, and primary health problem, and varying experiences with employment to capture heterogeneity among VRP participants. The collection of data stopped when we have reached a rich variety about IRP participant’s experiences [27, 28].

Recruitment was conducted by a coordinator, who contacted six potential participants. One interview participant was contacted personally by the first author (LNA) during an observation of a physical training session. Interview participants were recruited stepwise and the collection of data stopped when we had a rich variety of responses. All participants (*n* = 7) were sent written information and written informed consent was obtained (n = 7). Confidentiality was secured by presenting participants under ID (Table 1). All participants who were sent written information agreed to participate.

**Table 1:** Interview participant characteristics

Age group (years)	ID (sex m/f)*, time in IRP until now	Types of previous employment	IRP activities**
20–29	Participant 1 (m), 3 months	Never had paid work	Coordinator meetings (2–3/week) Relaxation therapist consultations Psychologist consultations Substance abuse consultations Course “Exercise and Motion”
30–39	Participant 2 (f), 2 years	Unskilled work	Coordinator meetings Relaxation therapist consultations Course “Exercise and Motion” (planned) Psychiatrist consultations (planned)
40–49	Participant 3 (f), 2.5 year Participant 4 (m), 1.5 years Participant 5 (f), 1 year	Manager Skilled work Unskilled work	Coordinator meetings in person or per phone Relaxation therapist consultations Course “Exercise and Motion” Course “Coping with everyday life” Education Internship (planned)
50–59	Participant 6 (f), 1.5 year Participant 7 (f), 0.5–1 year	Skilled work	Coordinator meetings Course “Exercise and Motion” Internship Pension applied for but refused

* Interviewed participants had been assigned to IRP for two to five years max

** Individual participant not offered all mentioned activities

*m* male, *f *female, *IRP *Interdisciplinary rehabilitation programme

Interviews were individual face-to-face interviews (duration: 44–145 min) and they were conducted from September 2016 until August 2017 by the first author (LNA). The first author (LNA) is an experienced interviewer. Depending on the participant’s preference, interviews took place either in the participant’s private home (*n* = 3) or in a designated room in the municipality social service office (*n* = 4). Interviews were audio-recorded and subsequently transcribed verbatim. Subsequently, participants were invited to read and comment on the accuracy of the transcription. Two participants accepted this offer.

All interview participants gave informed written consent to participate in the interview part of the study (not applicable for the quantitative part).

#### Interview guide

The semi-structured interviews were conducted using an interview guide as a flexible framework for questioning and dialogue with participants. The interview guide was developed based on the overall aims of the study [12] and themes included were: a) everyday life, b) the potential benefits of IRP, c) the development of work ability, d) relation between participant and coordinator, e) the impact of IRP, f) participants’ hopes and beliefs, their worries and anxieties. Interview questions in everyday language were developed, and each interview started with a short briefing about study aims and with an opportunity for the participants to describe themselves (age, residence, years in IRP etc.).The interview guide allowed for a conversational dialogical communication, though it focused on the overall aims of the project [12, 29]. Questions were open-ended and follow-up questions were asked if clarification was needed to enable in-depth descriptions from the participant [29].

#### Interview analysis

The qualitative data material from interviews was analysed using directed content analysis, a qualitative method that uses a pre-determined framework to guide analysis [30, 31]. “Stressful life events” and the five constructing elements of wellbeing in the PERMA model was used as predetermined coding framework (six themes).

Data was coded using “stressful life events” [15, 20, 21] as one predetermined theme and the five elements from the PERMA model was used [19, 22] as predetermined themes. Generating the theme “Stressful live events” data were also categorized under sub-themes separating out the content relating to either pain experiences or distress, depression and anxiety [30, 31].

After the coding process and sorting out the text elements in the predetermined themes, themes were reviewed for usefulness. The headings in the results section (Table 2) represents the predetermined thematic elements (stressful life events and the five thematic elements from the PERMA model). When reviewing themes, the thematic elements “Meaning” and “Accomplishment” were combined and presented under one heading.

**Table 2:** Overview of themes and subthemes

	Headings in the results section
	• Stressful life events - Painful experiences - Distress, depression and anxiety
*PERMA categories*	
***P****ositive Emotions*	• Positive emotions - how IRP-activities positively impacted wellbeing
***E****ngagement*	• Appreciation of engagement and co-decision
***R****elationships*	• Relationships
	- IRP and coordinator
	- Dispelling loneliness, sharing thoughts
***M****eaning* ***A****ccomplishment*	• Meaning and optimal functioning

First, the transcripts were examined, read and re-read to allow familiarization and to chart content relevant to developing an understanding of citizens’ wellbeing and participation in IRP. Categorization was deductive, but coding was flexible with new codes being added and existing codes being modified to allow new subthemes to emerge (LNA coded transcripts and content discussed with MJS and KKR) [30, 31]. Themes were named and written up (LNA, MJS and KKR). This approach contributed to an in-depth understanding of how citizens experienced life events and participation in IRP and how they were supported by the programme.

Quotations from the participants were used to give valuable insight but also evidence to the data collected from semi-structured interviews of IRP participants. The quotes illustrate and support the claims made by the researchers. All quotations used in the present paper were translated from Danish to English by a professional translator [32].

### Procedure for quantitative longitudinal survey

Questionnaires were sent via postal mail to all citizens assigned to IRP, initially at baseline and then at follow-up after one year. The questionnaire was designed to give an impression of the participants as regards their sex, age, height and duration of participation in IRP, and asked standardized questionnaires to describe self-reported general pain, distress, anxiety and depression, loneliness, physical fitness and physical activity.

In February 2016, a postal questionnaire was sent to all citizens who had been assigned to IRP during the previous year in Sonderborg Municipality and subsequently to all newly assigned citizens from February 2016 to March 2017. Two weeks later, a telephone reminder was sent to those who had not responded. Four weeks after baseline, the questionnaire was re-sent to non-responders. One-year follow-up questionnaires with the same questions were sent between February 2017 and March 2018 to all citizens who had returned the first questionnaire. If this was not returned a second letter with the same questionnaire was sent.

#### Measures

*General pain* was measured on a Visual Analogue Scale (VAS). The scale measures pain during the last seven days and uses a 100 mm VAS anchored with ‘no pain’ at 0 mm and ‘worst imaginable pain’ at 100 mm [33].

*Distress, anxiety and depression* was evaluated using the Patient Health Questionnaire-4 (PHQ-4), which is a 4-item screener for anxiety and depression [34]. It combines the Patient Health Questionnaire-2 (PHQ-2) and the Generalized Anxiety Disorder-2 (GAD-2) to give a measure of overall distress [34]. Responses are provided on a 4-point Likert scale ranging from “not at all” to “nearly every day” [34, 35]. The PHQ-4 total score ranges from 0 to 12, with categories of psychological distress being: None (0–2), mild (3–5), moderate (6–8) and severe (9–12). The anxiety subscale is calculated as the sum of items 1 and 2 (score range, 0 to 6) and the depression subscale as the sum of items 3 and 4 (score range, 0 to 6). On each subscale, a score of 3 or greater is considered positive for screening purposes [34].

*Loneliness* was assessed with the Three-Item Loneliness Score (T-ILS) [36]. Answers were recorded using a 3- point Likert scale with the response options “Hardly ever”, “Some of the time”, and “Often” [36]. A T-ILS sum score ranging from 3 to 9 was calculated by totalling the sum of all three answers. A high score indicates a high degree of loneliness. Based on previous studies, participants scoring 7 or higher were considered to be lonely [37].

*Physical fitness* was evaluated using a numeric rating scale with illustrations and verbal anchors for the extremes for five components [38]. One question relates to each of the five components: aerobic fitness, muscle strength, endurance, flexibility and balance. “How would you rate the following components of physical fitness compared with others of your own age and sex?”. Answers were recorded on an 11-point numeric scale ranging from weak to strong or poor to good [38, 39]. The measure has previously shown moderate to good validity in a large cohort and has been deemed a suitable alternative when objective measures are not feasible [39].

*Physical activity* was measured with a single question “This question is about how much you move and exert yourself physically during your leisure time” from the Saltin-Grimby Physical Activity Level Scale. The answer was recorded on a 4-point Likert scale measuring a range from almost completely passive to strenuously active [40–42].

#### Statistical analysis

General pain, PHQ-4, T-ILS and physical fitness: As the data were not normally distributed, the change from baseline to follow up was assessed using a one-sample Wilcoxon signed rank test. The proportion of participants with positive screening results for distress, anxiety and depression (T-ILS sum score and subscales) and physical activity were reported using frequencies and percentages. The change from baseline to follow up was assessed using the chi-squared test.

## Results

**Fig. 1:**
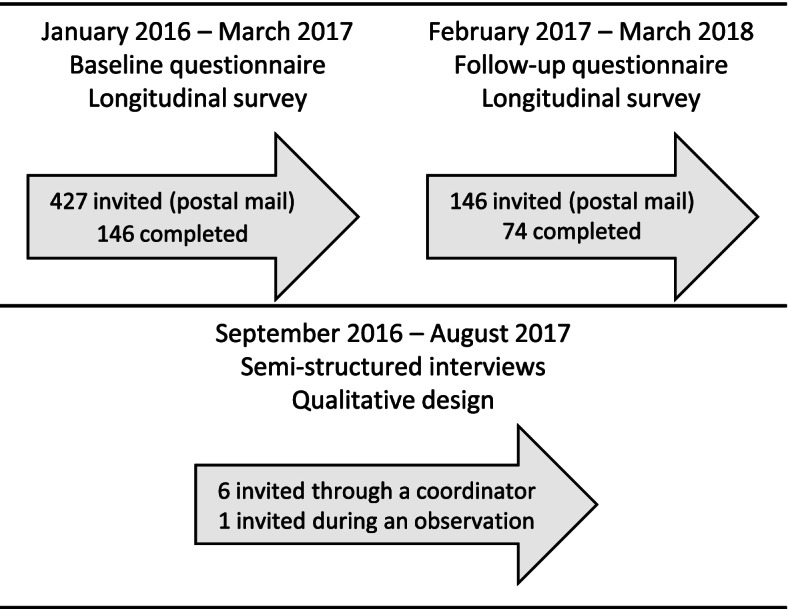
Inclusion and timeline for data collection

At baseline 427 IRP participants were invited, and the proportions were 146 (34%) men, 274 (64%) women and for 7 were sex not recorded. The baseline survey was completed by 104 women (71%) and 42 men (29%) (response rate of 34%). The proportions of sex were equivalent among completers and non-completers, see Fig. 1. Their median age [IQR] was 43.5 [31.0–50.0] and the median number of months participating in IRP [IQR] was 7.6 [1.2–14.0]. At follow up, 74 persons (51%) responded to the questionnaire. Follow-up responders were older than non-responders (mean difference of 3.9 years, *p* = 0.04), and more often had one or more children living at home (*p* = 0.031).

### Stressful life events

#### Painful experiences

Pain experiences from previous life events and chronic pain conditions were described by participants as causing stressful life events that impacted on their ability to function:

*So I have had problems with my back, and I can’t count the number of times I’ve had to get hold of the doctor on call and could hardly get out of my bed. (participant 7)*

*It was after I had my son, there’ve been so many things, because there’s always been something wrong (...) so I get pains in my neck and shoulder, some arthritis. (participant 5)*

Based on the self-reported questionnaire data (Table 3), a relatively high general pain intensity (median of 63 mm) was reported. The focus in the IRP was on improving participants’ functional abilities rather than decreasing pain. This was reflected in the follow-up survey, which showed relatively similar pain intensities at the two time points.

**Table 3:** Baseline and follow-up scores for general pain, T-ILS and PHQ-4

	Baseline *n* = 135	Follow-up *n* = 70	*p*-value
General pain, (100 mm VAS) median [IQR]	63 [31–84]	69 [25–85]	0.80
T-ILS, median [IQR]	5 [4–7]	6 [4–8]	0.50
PHQ-4 total, median [IQR]	8 [5–10]	6 [3–9]	0.07
PHQ-4 anxiety subscale, median [IQR]	5 [3–6]	3 [2–5]	**< 0.001**
PHQ-4 depression subscale, median [IQR]	3 [2–5]	3 [1–5]	0.46

IQR = Interquartile range; n = number

#### Distress, depression and anxiety

Experiences related to working life were described by some participants as causes for stress. Both participant 3 and 6 described their jobs as more and more exhausting and found it led to depression. Expanding on this, participant 3 described the challenge of balancing work and family obligations as pressure that brought on stress and the feeling of not being present in the moment:

*I felt I had a stop-watch up my backside all the time. It was living in the moment in the sense that now times up (clap), and now time’s up (clap), it was almost like interval training, constant time pressure, and I felt like a top spinning on its head. (participant 3)*

For those with job experience, their workload and their managers’ handling of difficult situations were described as important for the participants’ ability to cope with job-related stress. Participant 6 described how her work-related self-esteem progressively decreased, and how she felt after she was fired from her job:

*I loved being at work, I was good at my work, but it wasn’t there anymore. I would think, “Now I simply can’t sink any lower,” I had lost my job, my house, my boyfriend, and I was massively in debt. (...) “if a tree comes up now, you’ll drive into it”, and I’m sure I would have done it. But no tree appeared , there was a roundabout instead, and so I drove home to my parents and said: “I got to get to a doctor now”, and then I could let go (...) all I did those first couple of years was sleep. (participant 6)*

Survey results support the participants’ reports of stress and low levels of wellbeing. A large proportion of participants report moderate (27%) or severe (38%) distress. Of these, 66% scored three or more on one of the subscales indicating either anxiety and/or depression at baseline (Table 4). At one-year follow-up a significant development was seen when screened for depression; only one out of four participants were depressed at follow-up compared to two out of three at baseline (Table 4).

**Table 4:** Frequency and proportion of PHQ-4 screening categories (distress, anxiety and depression subscales) and T-ILS scores indicative of loneliness

	Baseline	Follow-up	*p*-value
PHQ-4			
Distress, n (%)			
None	20 (13.7)	15 (10.3)	0.18
Mild	26 (17.8)	17 (11.6)	
Moderate	39 (26.7)	17 (11.6)	
Severe	55 (37.7)	22 (15.1)	
Missing	6 (4.11)	75 (51.4)	
Anxiety, n (%)			
Yes	97 (66.4)	40 (27.4)	**< 0.001**
No	45 (30.8)	31 (21.2)	
Missing	4 (2.7)	75 (51.4)	
Depression, n (%)			
Yes	96 (65.8)	37 (25.3)	**0.002**
No	45 (30.8)	34 (23.3)	
Missing	5 (3.4)	75 (51.4)	
TILS > =7, n (%)			
Yes	52 (35.6)	26 (17.8)	**< 0.001**
No	91 (62.2)	45 (30.8)	
Missing	3 (2.05)	75 (51.4)	

n = number

Over a one-year course of the IRP, we observed a significant decrease in the number of participants with positive scores for both depression and anxiety (Table 4). In the interviews, participant 1 described how assistance from a psychologist taught him how to cope with previous stressful events and negative beliefs: “*That brought the realisation that I shouldn’t be so depressive, that I should run myself down*.*”* Likewise, participant 3 described how sessions with a psychologist taught her a ‘time-out’ strategy to handle situations that threatened psychological distress.

Participant 5 described how sessions with a relaxation therapist taught her to handle acute anxiety symptoms, and how the municipal coordinator supported her in being able to move around without being dependent on others.

#### Positive emotions - how IRP activities positively impacted wellbeing

Participants developed higher levels of wellbeing. They described how various IRP-activities contributed to development of wellbeing because the activities gave them a better physical capacity. Participant 5 described how her personal care situation has developed, because she gained better physical capacity. Participant 6 highlighted the significance of individualized physical activity sessions for changing her attitude towards the importance of physical activity for wellbeing:

*If they had told me, you’re going to have to go to training next year, I would have said I’m not bloody well doing that for a whole year. But if things developed well, then you’ll be allowed to continue, and that I think is really amazing (...) from being a lump where there weren’t any muscles left, she (the physiotherapist) really got my body working again, now I keep it going myself with the things she taught me. (participant 6)*

In line with participants’ personal accounts of limited physical capacity at the outset of the IRP, the survey baseline results indicate that participants generally scored low on questions relating to physical fitness and indicated an overall sedentary lifestyle (Table 5).

**Table 5:** Baseline and follow-up scores of physical fitness and physical activity in participants

	Baseline	Follow-up	*p*-value
Physical fitness	(*n* = 146)	(*n* = 73)	
Aerobic fitness, median [IQR]	3 [2–4]	3 [2–5]	0.77
Muscle strength, median [IQR]	4 [2–6]	3 [1.5–5]	0.68
Endurance, median [IQR]	3 [1–5]	3 [2–5]	**0.03**
Flexibility, median [IQR]	3 [2–5]	4 [2–6]	0.25
Balance and coordination, median [IQR]	4 [2–5]	3 [2–6]	0.71
Physical activity	(*n* = 145)	(n = 73)	
Almost sedentary, n (%)	54 (37)	23 (32)	0.81
Light activity, n (%)	53 (37)	31 (42)	
Moderate activity, n (%)	31 (21)	16 (22)	
Strenuous activity, n (%)	7 (5)	3 (4)	

IQR = Interquartile range

Over time, we saw only very small increases in physical activity, but we observed a significant improvement in endurance (Table 5). This aligns well with participants’ experiences of having developed more physical resources in cooperation with their coordinator. Participant 2 explains “*I had a good mentor who pushed me with a gentle tough in the direction of being able to walk a good distance and at speed”.*

Participant 3 experienced that participation in an IRP course had taught her about the relationship between physical exercise and stress, and how exercise improves wellbeing: “*The greater your knowledge, the more you understand. I’ll never leave off training, it’s so important for me to train”.*

### Appreciation of engagement and co-decision

In general, participants experienced that involvement in decisions about IRP activities facilitated their motivation for work-related activities and internships. Participant 5 described how she had been involved in decisions about progression in activities and how it was she who decided she was ready to start in an internship.

Participant 3’s development is one example of how participants in individualized ways described how interest in physical activity was sparked by an appreciation of its positive effect on wellbeing:

*I have never been a sports freak (...) now I am totally dependent. I started by just training physical training, and ‘Hey, man’ I can run, I got hooked on it. It was a revelation to see the physical effect of me becoming happy. (participant 3)*

Participant 3 went on to describe how participation in IRP slowly changed her way of engaging with her children. She experienced an improvement in her ability to concentrate, and learned to be aware of engagement in the here-and-now situation:

*I have got good at living in the moment (...) ‘Holy’ I have been so out of it, no good to anyone. That’s where I can feel the difference, I am very attentive – multi-tasking, forget it, I simply can’t do it anymore. (participant* 3*)*

Activities in IRP did not only facilitate participants’ engagement in activities. Participant 3 explained how she learned to balance her desire for increased wellbeing with developing her work ability, and described how the coordinator assisted her in building up her work ability with a realistic progression in work load:

*I have to try myself out as regards my work. I have got my qualifications (...) now I have to get it to balance out ‘what I am able to do’. (...) the risks of ending up down there again, they are there if give it everything I’ve got without a coordinator being there to say: ‘No, that twice three hours, now stop’. (participant 3)*

### Relationships

#### IRP and coordinator

Social relations impacted on participants’ development. Participant 3 described how going to IRP gave her a feeling of being supported and accepted as she was. The coordinator made her life story an integral part of her individualized IRP:

*I landed on the floor but I arrived in a transit lounge (= IRP) that could focus on the fact that you have a good and exciting life story, there’s something to grasp hold of. For a while, I couldn’t get up off the floor, what she (the coordinator) told me gave me optimistic motivation, enough to keep at the back of your mind that I wasn’t completely on the scrapheap. (participant 3)*

Overall, participants felt they were met with recognition and acceptance from others in IRP. Participant 2 told us how her collaboration with the coordinator was marked by acceptance, which was reflected in individual consideration of when and how starting internship. Participant 3 experienced how acceptance of her life situation and health issues by the IRP professionals helped her accept her route to future employment:

*I’m not good at boxes, but in this resource programme they really have said, yes, we can see that (...) My resources are there, there are just not in a measurable box. The council had to think outside the box because she (the coordinator) said that’s not you, not at all (about the list of internship places). (participant 3)*

Personal relations with the coordinators were described by participants as important for the development both of work skills and, of the social skills that were important prerequisites for employment. Participant 1 described the impact of personal interaction with his coordinator:” *(…) it helps me to be more social.”* While a trusting relationship with a coordinator who acts as a support and guide in learning self-management was explained by participant 5. She described, for example, the experience of doubting whether she should participate in an experiment proposed by her psychologist, and her coordinator offered reassurance by phone about decision-making:” *It is as though she (the coordinator) is the adult, she has to say, ‘But that is what you have decided”.*

#### Dispelling loneliness, sharing thoughts

More than a third of participants in the survey scored seven or more at baseline on the T-ILS, which indicates loneliness. One year later, this was significantly reduced to less than one in five, with men showing the largest reduction (Table 4). These results were supported by participant 3’s experience of loneliness before IRP participation. At that time, she did not have a job and she was not registered in the municipal system:

*To feel that you don’t represent anything that anyone can relate to. I had cut myself off from pretty much everyone, it was very lonely, even though I had a large family. It was lonely even though I wasn’t lonely, for inside it felt very lonely and wrong. (participant 3)*

The absence of trusting relationships was highlighted by participants as important for their experience of loneliness. Relations with spouses or near relatives offered this type of support for some participants:

*Something that has affected me is that he (husband) has fought his own battles but also our battles. He could see that I wasn’t able to fight. He has been along to every interview. (...) He’s good with words, so he could say the things I couldn’t say, I just sat there like a grey mouse. (participant 3)*

In addition to the fellowship created with their coordinator, other participants found that s/he provided them with the opportunity to share their thoughts. The personal interaction with the coordinator gave participants feelings of being cared for, which was significant for dispelling their feelings of loneliness, for example when interest was shown in their personal development, as described by participant 5: “*They were so positive, at times more stressed (=happy) than I was – yes, you could do it, yeay!”*

### Meaning and optimal functioning

Participant 3 described how she developed understanding and from that the possibility to progress and build a new and more balanced life:

*A lot of people say, if they have been down, that now I’m going back to being my old self (...) But you should never go back, for that was what made you ill. (participant* 3*)*

When participant 3 reflected on her progress, she realized not only had she found direction in her working life, but she had also developed a more meaningful life in general:

*I could contribute a whole lot at work, but that was all I could do, that was pretty pathetic. At that level I feel I have been enriched, I have really come a long way. (participant 3)*

In addition, participant 6 found that talks with her coordinator were conducive to finding a direction for her working life, and participant 5 described a significant sense of community with other IRP participants in physical activity sessions, which contributed to a sense of being happier: “*You can see how the others change, and that makes you happy”.*

Different ways of developing competencies to attain personal goals were described. Participant 4 learned how to balance work tasks without getting pain, while participant 6 described developing the ability to distribute her resources to master activities in an acceptable way that gave a sense of accomplishment: “*I am not as I was in the old days, I’ll never be that again, but I can certainly accept things as they are”.* Participant 3 has developed a sense of having mastered work-related activities successfully, because she has identified realistic job opportunities she is passionate about: *“I know my stuff, I just hadn’t realised what it was I was supposed to work with”.* Both experienced feeling better through mastering internships successfully. Participant 3 further elaborated that, based on her life values, her functioning as a human being had improved:

*It’s ok if I am to die – no, of course I hope I am not. If I had had to say goodbye when I was in tenth gear, that would have been sad, ‘cos then I hadn’t a clue about anything, or how you can live harmoniously and in touch with yourself. But I found the resources that give me a quality of life, and that’s what it’s all about, of course, giving life quality. (participant 3)*

## Discussion

The results from this mixed methods study, combining survey and interview data to investigate citizens’ experiences when assigned to a IRP in Denmark, indicate that as a whole these citizens were confronting pain as disrupting their wellbeing and functional ability. In addition, the participants’ experiences of work as regards job content, workload and working environment induced stress and had a bearing on their depressive feelings. In addition, some participants’ negative beliefs about themselves were experienced as being related to depression. Participants’ experiences of distress were supported by the survey results, which showed that more than one third of the participants experienced distress and a large proportion of them experience anxiety, depression and loneliness.

IRP participants’ descriptions of stressful life events and how they impacted on their health and wellbeing are consistent with findings from Cleland et al. [20] who have shown how significant life events e.g. health issues, the death of someone close, or relationship breakdown, cause a range of contradictory emotions and impact on physical health, mental health and wellbeing. Further, Cleland et al. [20] showed that getting a job or being promoted has positive effect on both physical and mental health as well as on wellbeing, and they underline the importance of employment for health and wellbeing [20, 43]. In that light IRP activities can be seen to offer the ability to build participants’ positive emotions through activities directed towards future employment [21, 22].

### PERMA

For many of the participants, the road to flourishing was paved partly by positive experiences deriving from physical training sessions. In the survey, IRP participants generally reported sedentary lifestyles and low physical fitness but, over time, an increase in self-reported endurance was observed. In the interviews, participants described positive training experiences and the building up of physical capacity not only as a means to better physical health but also as a form of stress management and a source of positive feelings [22].

Positive emotions are one element out of five that underpin the construct of flourishing and the PERMA model [19, 22]. Flourishing is about learning how to lead a good life. In this view, physical activity may be significant both for mental and physical health, and it is in general recommended to be physically active for example from the WHO [44]. Positive emotions and physical activity should be seen in combination where they each influence each other and can reinforce each other. Physical exercise has shown a beneficial effect on a range of mental health conditions [45–48], and works through both positive physical and psychological mechanisms. Physical exercise buffers against stress and stress-related disorder, which may be one potential explanation for the positive emotions evoked in the interview participants [49]. Interpreted in the light of The Broaden-and-Build Theory [50], the participants’ positive attitudes to and experience of physical exercise led both to an increased capacity for physical work and, as a consequence, to a better chance of entering the work force and a broadened repertoire of psychological skills and resources for managing stressful situations.

Population studies have shown that flourishing is inversely related to levels of functional limitation [51]. Therefore, it may be inferred that IRP activities increased flourishing through the positive experiences that in turn increased participants’ ability to function. In this process, self-acceptance is important for the development of positive emotions and wellbeing [50, 52]. Self-acceptance is the individual’s acceptance of all his/her attributes, whether they be positive or negative [52]. In individuals with functional limitations, self-acceptance has been hypothesized as being an important construct, because it enables individuals to view themselves positively and to be better equipped to cope with their limitations and to establish healthy behaviours [52].

IRP participants felt they were being met with acceptance from others. They felt the backing of IRP professionals, who supported participants in their acceptance of past and present situations, which enabled them to view themselves more positively. Self-acceptance and increased flourishing were accomplished through recognition and acknowledgement of individual strengths by others, which in turn nourished positive emotions and motivation for rehabilitation and future employment [50, 52–54]..

Participants found that IRP activities taught them to focus on the present moment (e.g. when relaxing or socializing with family) and their engagement in co-decisions about IRP activities stimulated their feelings of curiosity and development of interest for work-related activities [50, 54]. Interest has previously been suggested as an important driver for building personal skills and wellbeing [54]. Increasing interest can be interpreted as an important personal quality promoting flourishing, an important contribution to developing participants’ capacity to work [19, 54] and an opportunity to cultivate the best version of themselves in relation to working situation [18, 50, 54].

Compared to both the general Danish population and populations in 15 other countries, IRP participants reported high levels of loneliness at baseline with some improvement at follow-up [55, 56]. The participants’ accounts of how relationships (e.g. with IRP coordinator, health care professionals or a spouse) helped the participants in their development towards improved work skills were in line with the survey findings. Trusting relationships with healthcare professionals have previously been shown to be significant for a patient’s journey towards healing [13]. In addition, our study results indicating that, sharing thoughts is supportive are in line with previous findings from Roessler et al. [57] about the significance of supportive relationships for wellbeing and health.

Participants described how their individual life stories (past/present/future) were taken into account when the individual IRP programme components were planned, which led to a sense of meaningfulness and to a sense of connectedness with others [18, 22, 58]. Previous studies have highlighted the importance of a good fit between a new job and a person’s interests and abilities for successful re-entry into the work force [59, 60]. Having the experience of responding relationships can change behavior [57, 61] and these emotions supported participants in finding direction for their working life [59] as well as shaping flourishing conditions for them [21].

Through the IRP activities, participants learned how to manage their personal resources in order better to cope with life situations, and in so doing they achieved a sense of personal accomplishment. A sense of accomplishment was also generated when participants were given opportunities to find types of work that were meaningful and at the same time realistic, given their life story [22, 62]. Giving support and space to find work and other activities that match participants’ individual values can be regarded as significant factors for promoting feelings of accomplishment for IRP participants [19].

### Methodological reflections and limitations

The positive long-term development of anxiety, depression and loneliness should be interpreted with caution, as we had a low response rate at follow-up, and selection bias cannot be ruled out, even though acceptable response rates from 25% have been suggested [63, 64]. The overall positive development may be as a result of the most resourceful participants being the ones to answer the questionnaire. However, the responder analysis showed no significant difference between responders and non-responders in baseline levels of anxiety, depression or loneliness, and the qualitative results highlighted the positive development during IRP. To ensure a higher follow up rate and potentially reach the participants with the fewest resources, future evaluations should consider surveying the participants by means of direct phone or face-to-face contact.

Regarding loneliness, a conservative approach to estimating loneliness was used. Because there is no consensus about estimates for loneliness, we have used the limit of 7 proposed by Lasgaard et al. [55] to estimate loneliness. Another study [65] on older adults has used a minimum score of 6 which would have given a higher proportion of participants experiencing loneliness in our study.

In future studies it may be relevant to explore issues relating to specific subgroups, such as age or sex. Our empirical data allowed to make descriptions by gender from the quantitative data however our empirical data did not allow to discuss on sex subgroups in relation to the research questions.

We collected data in the qualitative and quantitative parts of the study concurrently [12]. It was useful and provided nuanced insights to answer study aims. Using other types of mixed methods research designs and doing data collection and analysis in other ways may offer valuable insights and may be useful for future studies. Our conclusion does not refer to an existence of an objective truth but gives knowledge and understanding about participants assigned to IRP and their personal development.

Merging data is challenging and using visual aids as joint displays may have provided a beneficial structure for the integration of data in the present study [66, 67]. We used a predetermined framework was used to provide structure for the analysis and section with results. We are aware that there may be limitations associated with using a framework. The semi structured interviews allowed interviewees to provide their descriptions both critical and non-critical. We used a transparent and well-known approach for our analysis, namely the directed content analysis. We used predetermined thematic elements, but coding was flexible and inductive to allow new subthemes to emerge. Taken together with our study aims not aiming for an objective or generalised truth but for descriptions, exploring impacts of life events and understanding of IRP participants’ development towards future employment the study has provided knowledge about these aims. As mentioned, it may not be the only truth, but results will be useful knowledge for future designing of IRP’s.

## Conclusion

IRP participants experience high general pain levels and a large proportion of participants experienced distress, depression and anxiety. Various stressful life events relating to both physical health and work life are described by participants as significant for their wellbeing. IRP activities can build participants’ positive development in individualized ways through activities directed towards future employment. Features that participants developed through participation in IRP activities relate to self-acceptance, acceptance from others, physical capacity, psychological resources, and learning to balance engagement to cultivate the best version of themselves and the development of interest as a driver for wellbeing and future employment possibilities. In the future it may be worth to investigate how physical activity impact not only physical capacity for IRP participants, but also positive emotions and stress management.

## Data Availability

The collected datasets used for analysis in this paper will not be publicly accessible to preserve the privacy of participants. However, they will be available from the corresponding author on reasonable request.

## Abbreviations

GAD-2: Generalized Anxiety Disorder-2
IRP: interdisciplinary rehabilitation programme
IPS: Individual Placement and Support
IQR: Interquartile rang, n = number
PHQ-2: Patient Health Questionnaire-2
PHQ-4: Patient Health Questionnaire-4
T-ILS: Three-Item Loneliness Score
VAS: Visual Analogue Scale
